# The Influence of Surface Texture of Elements Made of PA6-Based Composites on Anti-Graffiti Effect of Paint Coating

**DOI:** 10.3390/ma17091951

**Published:** 2024-04-23

**Authors:** Adrian Mróz, Maciej Szymański, Paweł Koch, Marek Pawlicki, Artur Meller, Robert Edward Przekop

**Affiliations:** 1 Mechanical Engineering Institute, Collegium Mechanicum, The President Stanislaw Wojciechowski Calisia University, 4th Nowy Świat Street, 62-800 Kalisz, Poland; m.pawlicki@uniwersytetkaliski.edu.pl; 2 STER Institute Maciej Szymański, 1st Ster Street, 62-080 Swadzim, Poland; ster@ster.com.pl; 3 STER Sp. z o.o., 1st Ster Street, 62-080 Swadzim, Poland; pawel.koch@ster.com.pl; 4 Pratt & Whitney Rzeszów S.A., 120th Hetmanska Str., 35-078 Rzeszów, Poland; 5 Faculty of Mechanical Engineering, Poznan University of Technology, 3rd Piotrowo Street, 60-965 Poznań, Poland; artur.meller@doctorate.put.poznan.pl; 6 Faculty of Chemistry, Adam Mickiewicz University in Poznań, 8th Uniwersytetu Poznańskiego, 61-614 Poznań, Poland; robert.przekop@amu.edu.pl

**Keywords:** polyamide, protective organic paint coating, anti-graffiti effect, roughness, texture

## Abstract

The aim of the work was to investigate the influence of the surface texture of composite based on PA6, intended for wet painting, on the stability of the colour and gloss parameters. The stability of the paint coating was required to be maintained despite exposure to mechanical stress resulting from attempts to manually remove graffiti stains. The study examined the influence of surface texture on the effectiveness of cleaning. In the case of painted surfaces from which graffiti stains were effectively removed, the roughness, colour parameters and gloss of the paint coating were measured. During the research, it was found that roughness after painting decreased to the value of Ra < 2.00 µm meets aesthetic expectations and at the same time ensures the effective removal of graffiti stains. For this surface, there were no negative effects of the mechanical impact on the textures or quality parameters of the coating as a result of manual graffiti removal. As a result of the conducted research, the recommended maximum values of roughness and textures of the surfaces to be painted were determined in order to ensure a sufficiently low amount of work necessary to effectively remove traces of graffiti.

## 1. Introduction

Acts of vandalism in the form of graffiti are a problem for the authorities and residents of many cities around the world. The need to protect surfaces against acts of vandalism in the form of graffiti applies, in particular, to buildings, including those characterised by high historical and cultural values [1]. The costs associated with removing graffiti run into millions or billions of euros, depending on whether the scale of the phenomenon concerns countries or individual cities [2]. Another type of objects exposed to devastation are public spaces, which include the interiors of public transport vehicles such as buses, trams and trains [3]. In accordance with the DB information service, Deutsche Bahn AG noted about 14,000 cases of graffiti damage in 2015 [4]. Passenger space equipment, especially in public transport vehicles (e.g., seat bodies), is made of plastics and polymer-based composites. One of the most commonly used materials is PA6 reinforced with glass fiber (GF). The widespread use of this material is due to, among others, its high tensile strength and elasticity, good thermal properties and chemical resistance [5]. In the manufacturing process of bus seats, GF-reinforced PA6 composites are used for parts that are more flexible, which provide comfort (e.g., seat and backrest inserts are usually made of PA6-GF10), and ones that are more responsible for passenger safety (e.g., shells are usually made of PA6-GF30). Flame retardancy of the material is an increasingly required property (FR) [6]. PA6 and its composites are characterised by a good surface energy level and this is why they can be more easily painted than polypropylene. An analysis of the influence of the temperature conditions in a paint shop in the automotive industry (resulting in the overheating of painted thermoplastics) on the mechanical properties of the PA6-GF composite was presented in [7].

Removing graffiti using mechanical and chemical methods often carries the risk of changing the physical and chemical properties of the surface. This also applies to polymer-based composites. Therefore, it is vastly important to develop solutions that effectively protect the surfaces, as well as to select methods for removing graffiti stains that are relatively safe for the substrate material. Literature reports on the first documented attempts to develop anti-graffiti coatings come from the 1960s. At that time, in New York, an acrylic coating called Hyfdon 300 was used to protect public transport vehicles [8]. The idea of reducing the surface energy of the protective coating remains valid to this day [9]. Over the years and with the development of science, only the materials and technologies have changed.

In the case of polymer-based composite materials, the properties of moulded parts can already be modified at the stage of injecting the material into the moulds by introducing appropriate additives such as paraffin waxes and silicones [10,11]. The disadvantage of this approach is that in industrial practice, it raises problems due to the need to adhere to different technological regimes than the standard ones. In addition, structural changes may affect the properties of the moulded parts, which may result in the need to redesign parts to ensure the same safety conditions. Cost effectiveness is also worse, and mass modification is not justified in terms of protecting the material against the effects of vandalism. The use of fluorine polymers is also economically unjustified. Inexpensive and commonly available polymers or polymer-based composites can be processed. An example of such technology is direct fluoridation [12]. Unfortunately, the thickness of the modified layer is not impressive, ranging from a few nm to a few µm [9]. In the conditions occurring in the passenger space, this may not be sufficient.

In this context, a much more attractive solution is the use of wet-applied polyurethane paint coatings. This technology offers the possibility of applying coatings with a thickness of several dozen micrometres. The initial investment cost is significantly lower. In addition, wet-painting technology enables quick and inexpensive reconstruction of the coating in the event of its destruction. It is also advantageous that the paint coating can constitute a protective barrier against external factors, such as UV radiation [13,14] in the case of polymers or anti-corrosion in the case of metals. The anti-graffiti properties of polyurethane paints or acrylic paints are not provided by strong C-F bonds [15].

Anti-graffiti properties are related to surface energy and surface wettability. The hydrophobic and oleophobic effect is verified using contact angle measurements. It is assumed that a surface is considered hydrophobic when a water droplet creates a contact angle greater than 90° [16]. In the case of lipophobicity, it is difficult to determine the limit value because for different oils, and the value of the angle created by a drop on the same surface may be different. The effectiveness of graffiti removal should also be verified using measurement techniques that enable objective control of the colour and gloss parameters. This is important because the use of a more aggressive chemical agent may result in high graffiti removal efficiency. This phenomenon cannot go hand in hand with the destructive impact of this substance on the surface to which it is applied. Guidelines in this regard are included in the ASTM D6578-13 standard [17]. Moreover, the mechanical impact during graffiti removal should not lead to a change in the physical characteristics of the surface layer. The authors of article [15], while examining the impact of the use of various cleaning agents on the condition of organic anti-graffiti powder coatings covering aluminium tiles, additionally took into account the aspect of surface roughness. The research results confirmed that surface roughness has a significant impact on the effectiveness and ease of graffiti removal. Another interesting aspect of the relationship between hydrophobicity, roughness and transparency of acrylic coatings is presented in publication [18].

A review of the literature carried out by the authors of this article did not reveal any publications that examined the influence of the roughness of the protected surface against the effects of graffiti on the roughness value of the protective coating, and further on the effectiveness and ease of removing graffiti. The aim of this study was to investigate the influence of the surface condition (texture) of elements made of a composite based on PA6, intended for wet painting, on the stability of the colour and gloss parameters of the paint coating after a graffiti removal test (aerosol paint and xylene marker). It is expected that the research will enable the determination of recommendations regarding the maximum values of roughness parameters and textures of surfaces intended for painting so as to ensure a sufficiently low amount of work necessary to effectively remove traces of graffiti, and at the same time not lead to a destructive mechanical impact on the roughness peaks.

## 2. Materials and Methods

The test samples were produced using material injection technology, PA6 GF30 (TARNAMID T-27 GF30 FRV0, Azoty, Tarnów, Poland), into moulds. The injection process was carried out utilising a JM1000-C2 injection moulding machine (Chen Hsong Machinery Co., Ltd., Hong Kong, China) with a capacity of 1000 kg/h. The mould elements were made of steel 1.2738 (ISO 4957 [19]). The texture of the samples is the result of previous chemical texturing of the replaceable forming elements of the mould (Textures 1–4). The reference sample was a plate injected employing a mould whose forming component was not chemically etched (Textures 0). After conditioning (7 days), it was sent for painting. Preparation included the manual application of antistatic fluid and deionisation of the surface. The painting process was carried out in two steps: (1) the application of the primer and (2) the application of the final coating (1 layer) of paint based on acrylic resins with an anti-graffiti addition by means of a paint applicator (1.2 mm nozzle). The painted samples were dried in a dryer (according to the manufacturer’s recommendations). After drying, the painted samples were conditioned (T: 20–23 °C, RH: 40–70%, t: 7 days) to achieve complete curing.

Before commencing the main phase of research, a qualitative assessment of the samples was carried out. The assessment included visual inspection with the naked eye using a Colour Viewing Light XXL Professional light chamber (Deep Blue Technology Co., LTD., New Taipei City, Taiwan). The evaluation was performed using D65 lighting (daylight, 6500 K). The quality assessment also included verification of adhesion to the substrate in accordance with ISO 2409 [20] (cross-hatch method) using an Elcometer 107 Cross-Hatch Cutter (Elcometer, Manchester, UK) with a 6 × 1 mm cutter and ISO tape, as well as measuring the contact angle. Water contact angle analysis was performed by means of the sessile drop technique at room temperature and atmospheric pressure using a Krüss DSA100 goniometer (Krüss Scientific, Matthews, NC, USA) with a 5 µL and 10 µL deionised water drop, respectively, for static and dynamic characterisation (other parameters during dynamic characterisation: total measurement time of one drop: 60 s; frequency: 5 measurements per 1 s; dosing rate: 100 μL/min (from 0 to 5 μL) and 10 μL/min (from 5 to 10 μL). The basic investigations included measurements of the roughness and surface textures in addition to colour and gloss parameters (all carried out before and after applying the stains—graffiti). The roughness measurements (Ra, Rz, Rt, Rq) were carried out by means of a Nanoscan 855 Jenoptik (Hommel–Etamic, Bayeux, France) profilometer. The test parameters were Il = 4.80 mm, Vt = 0.50 mm/s, Ic = 0.80 mm. Before starting the roughness measurements, the correctness of the profilometer readings was verified with a master plate (Ra = 1.00 µm, Rz = 3.30 µm (RNDH 2, No. 231498)). Measurement of the colour and gloss parameters was conducted using a CM-36dGV spectrophotometer (Konica Minolta, Hong Kong, China) with D65 illumination and the CIE LAB scale as well as the quality control module of Colibri software v.3.8.12 (Konica Minolta), applying the following parameters: measuring geometry—diameter of the measuring diaphragm: 25.4 mm, d/8°, gloss measurement angle: 60°, T = 21 °C, HR = 55%. All the applied measurements (water contact angle, roughness, colour and gloss parameters) were repeated 7 times for each sample (the two extreme values, the lowest and the highest ones, were not taken into consideration).

The process of applying graffiti to the samples was performed using a marker containing xylene (XM), Industrial Paint Marker TO-450 (Toma Sp. z o.o., Przeźmierowo, Poland), and aerosol paint (SP), Hardcore Spray Paint—Negro Black (Montana Colours S.L., Barcelona, Spain). The graffiti removal process was carried out 24 h after the contamination was applied to the researched surface using (1) a dry cotton cloth (XM(D) and SP(D), where “D” means “dry”) and (2) a cotton cloth soaked in isopropyl alcohol (IPA) Contact IPA plus (AG TermoPasty, Sokoły, Poland) (XM(I) and SP(I), where “I” means “soaked in isopropyl alcohol”). The graffiti removal process was undertaken manually by the same operator in all the specimens. During the process, the stress was only controlled indirectly using analytical balance equipment to ensure the most repeatable conditions possible.

After the graffiti was completely removed, the colour parameters (ΔE coefficient) and gloss (G/Gi) were measured. In order to consider the trace of graffiti completely removed and the paint coating not negatively affected by the employed removal method, two conditions had to be met: ΔE < 2.00 and (G/Gi) ∈ <0.90–1.10>. The methodology for applying graffiti, removing it and assessing the effectiveness of cleaning was developed based on the guidelines contained in the ASTM D6578-13 standard (Method B, instrumental). The number of the measurements was the same as after the coating application.

Digital imaging of the painted surfaces and topographic assessment of the surface were performed utilising a VR-6000 optical 3D profilometer (Keyence, Osaka, Japan). Only representative samples were subjected to observation and measurements of the surface texture.

## 3. Results

**Figure 1:**
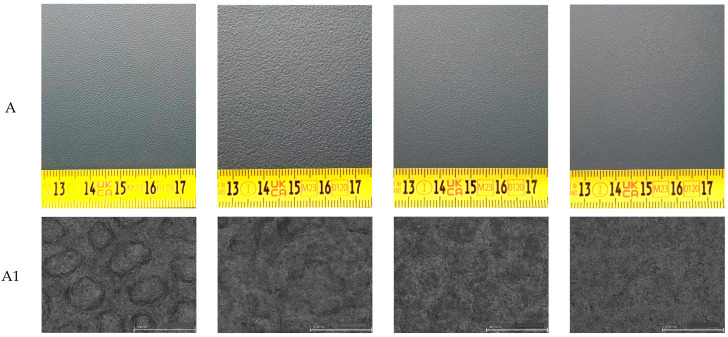

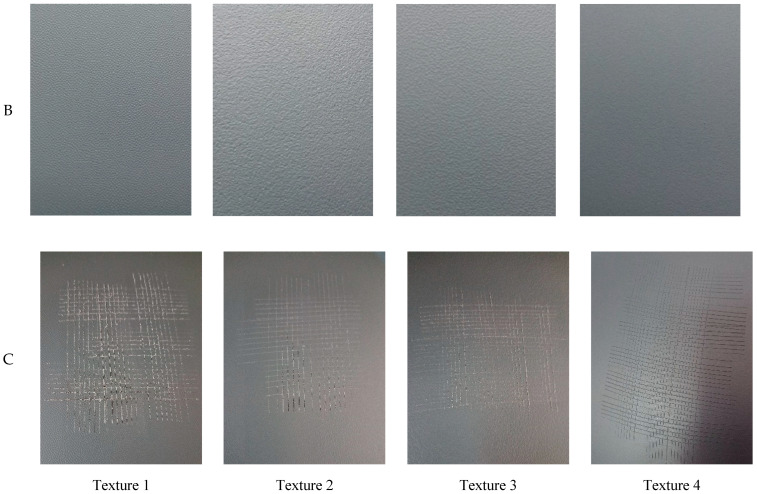
General view of studied surfaces: (**A**)—before painting ((**A1**)—digital image (mag. 120×)), (**B**)—after painting, (**C**)—adhesion test (observation conditions: D65 light).

A general view of the surfaces in D65 lighting conditions, before painting (A), after painting (B), and after testing the adhesion of the coating to the substrate (C), is shown in Figure 1.

Very good adhesion of the coating to the substrate was confirmed for all the investigated surfaces (Figure 1C). The results of the texture characterisation contact angle measurements revealed that a strong hydrophobic effect was confirmed for the paint coating, regardless of the substrate texture (Table 1). The wettability of the structured surface was significantly higher (by about 20°) than that of the smooth sample (texture 0–ref.).

The obtained results show that both the free surface energy of the coating and the texture (roughness) influence the wetting angle (from 105.83 up to 109.54°).

**Figure 2:**
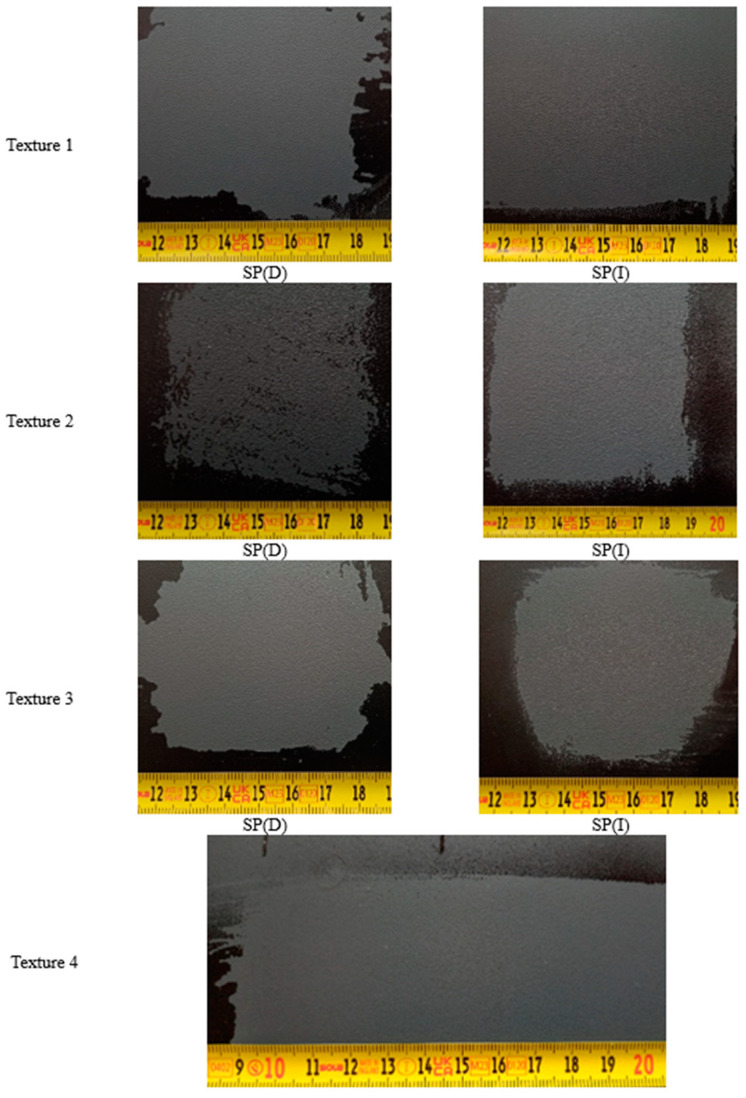
General view of researched surfaces after SP graffiti removal test, where (D)—dry cotton cloth and (I)—cotton cloth soaked in isopropyl alcohol (IPA) (observation conditions: D65 light).

The effects of SP graffiti removal are shown in Figure 2.

For most samples, the SP graffiti removal was effective. The exception was the surface that was characterised by the relatively highest roughness—Texture 2 (Ra = 5.155 ± 0.420 µm and Ra = 3.826 ± 0.432 µm, before and after painting, respectively). After the cleaning test, visible stain residues were observed in the roughness valleys.

**Figure 3:**
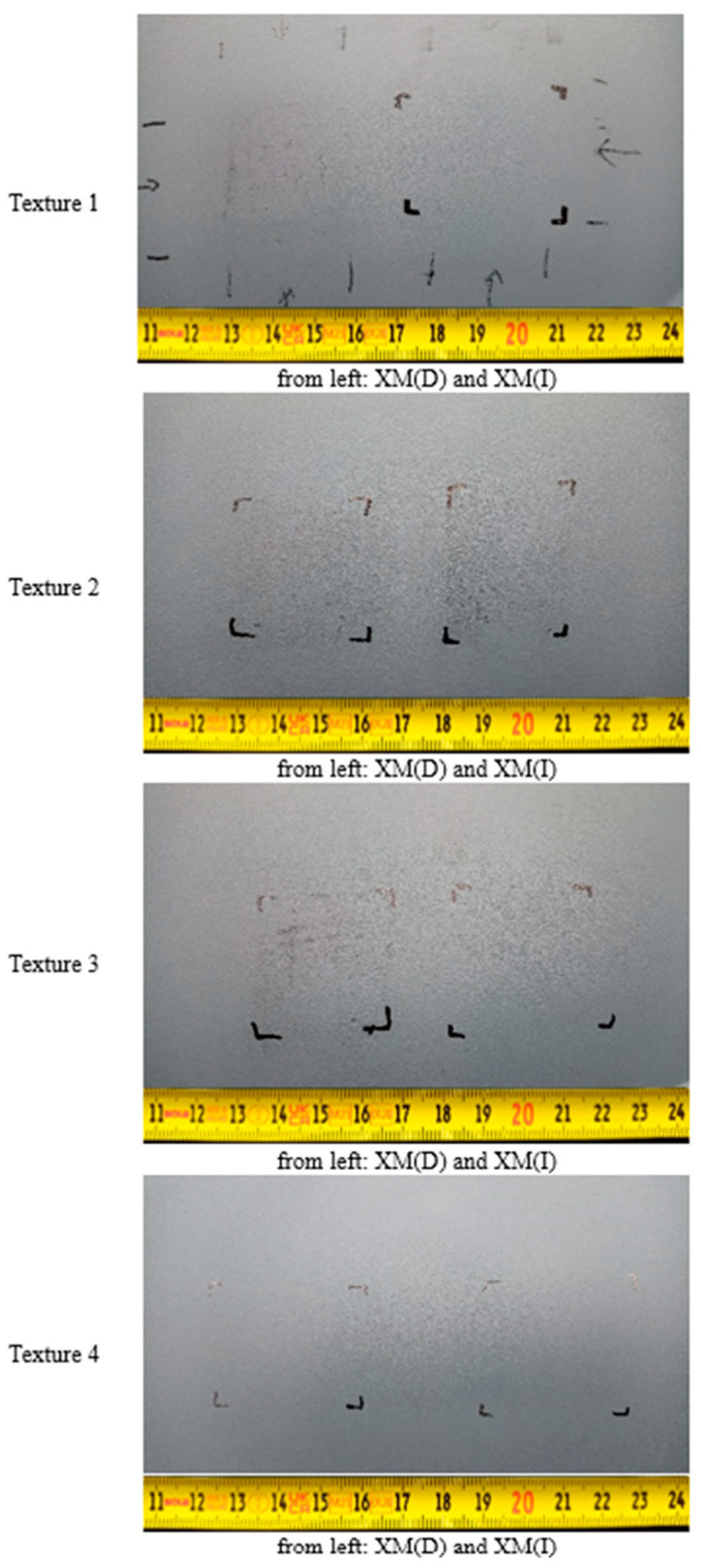
General view of studied surfaces after XM graffiti removal test, where (D)—dry cotton cloth and (I)—cotton cloth soaked in isopropyl alcohol (IPA) (observation conditions: D65 light).

The effects of XM graffiti removal are displayed in Figure 3.

**Figure 4:**
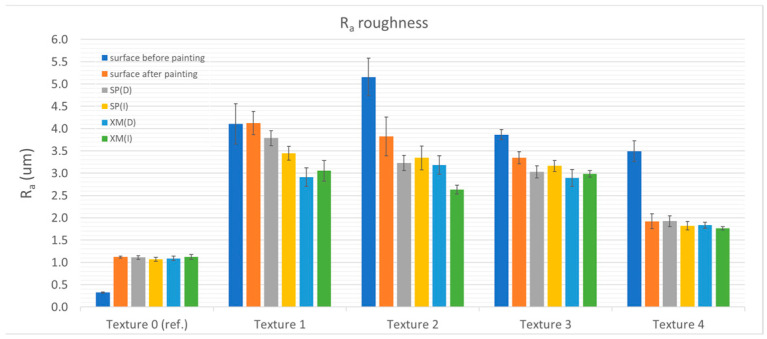
Ra roughness in relation to textures and surface condition.

**Figure 5:**
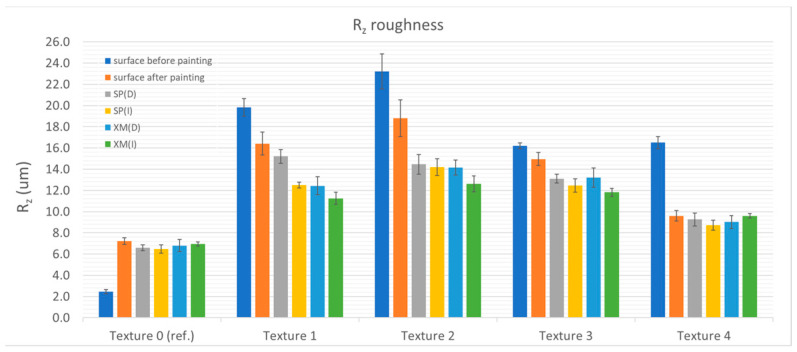
Rz roughness in relation to textures and surface condition.

**Figure 6:**
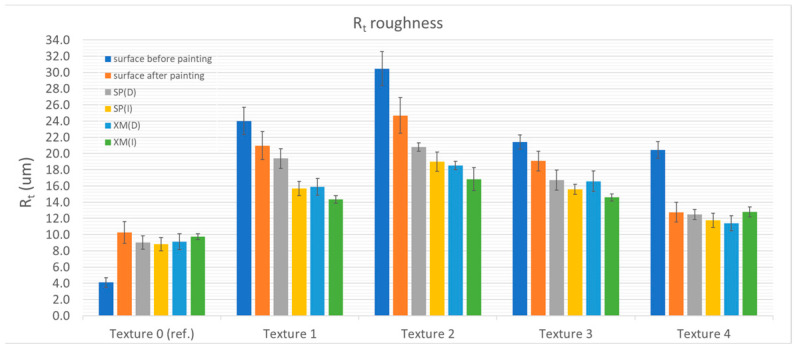
Rt roughness in relation to textures and surface condition.

**Figure 7:**
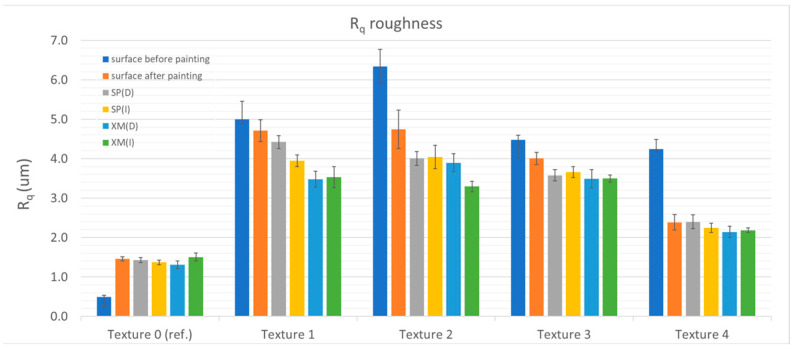
Rq roughness in relation to textures and surface condition.

The cleaning tests showed that removing XM graffiti with a dry cloth was possible only in the case of the reference sample (Ra = 0.324 ± 0.016 µm and Ra = 1.120 ± 0.020 µm) and the sample with the structure with the relatively lowest roughness—Texture 4 (Ra = 3.494 ± 0.231 µm and Ra = 1.922 ± 0.166 µm, before and after painting, respectively). For all the other samples (Textures 1–3), traces of graffiti were observed in the roughness valleys after testing. By using a cloth moistened with alcohol, the graffiti was completely removed from most of the studied surfaces (Textures 0, 1, 3 and 4). Figure 4, Figure 5, Figure 6 and Figure 7 show the results of the roughness measurements.

The cleaning tests showed that removing XM graffiti with a dry cloth was possible only in the case of the reference sample (Ra = 0.324 ± 0.016 µm and Ra = 1.120 ± 0.020 µm) and the sample with a structure with relatively the lowest roughness—Texture 4 (Ra = 3.494 ± 0.231 µm and Ra = 1.922 ± 0.166 µm, before and after painting, respectively). For all the other samples (Textures 1–3), traces of graffiti were observed in the roughness valleys after testing. Using a cloth moistened with alcohol, graffiti was completely removed from most of the investigated surfaces (Textures 0, 1, 3 and 4). Figure 4, Figure 5, Figure 6 and Figure 7 present the results of the roughness measurements.

For most of the researched samples that were textured (1, 2 and 3), significant smoothing of the paint coating surface was observed in the zone where cleaning was carried out. This phenomenon was found after removing the SP and XM stains. The exceptions to this rule were Textures 0 and 4, for which the roughness before staining was comparable to that found after removing the graffiti stains. The smoothing effect was caused by mechanical (shearing) impact on the roughness peaks. Moreover, it is significant that during the cleaning process it was noticed that higher surface roughness results in graffiti stains remaining in the roughness valleys. This causes the person removing the graffiti to instinctively start the cleaning process using greater force (pressing the cloth against the surface) and/or using a larger number of cycles (the cleaning process took relatively longer). As a result, the smoothing effect was greater. The cleaning effect using the alcohol-soaked cloth was worse than that of the dry cloth.

When comparing the roughness measurement results, another relationship was found. In addition to the fact that the effect of changes in the roughness resulting from cleaning is greater with the greater roughness of the surface before painting, it was noticed that in the case of the samples cleaned with the alcohol-soaked cloth, the decrease in roughness was greater than in the case of the samples cleaned with the dry cloth. The phenomenon of greater wear can be explained by the phenomenon of softening of the top layer of the paint coating as a result of exposure to isopropyl alcohol (regarding Textures 1 and 3). The smoothing of the surface made the surface brighter (increased gloss). Table 2 shows the results of gloss measurements as well as the results of calculations of the colour deviation (ΔE) and the relative change in gloss (G/G_I_, where G is the measured gloss and G_I_ is the gloss of the paint coating before applying the graffiti).

**Figure 8:**
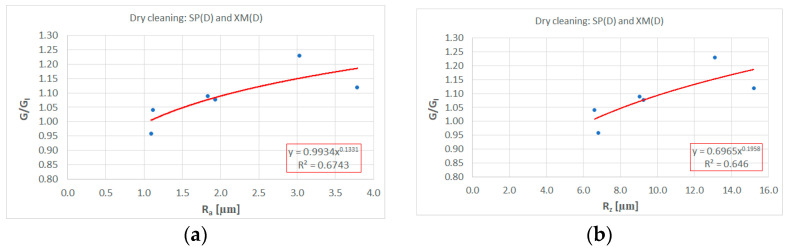

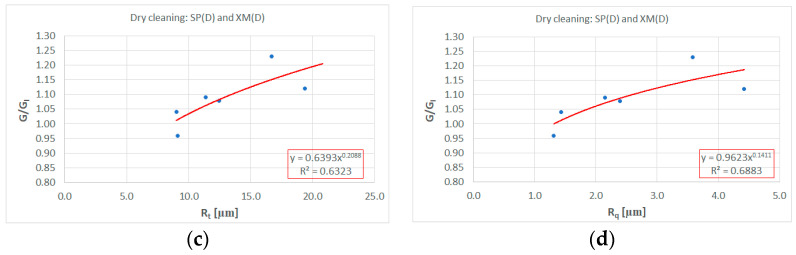
Roughness measurement values in relation to relative change in gloss after cleaning in dry condi-tions; (**a**) Ra; (**b**) Rz; (**c**) Rt; (**d**) Rq.

Figure 8 shows the values of the roughness measurements as a function of the relative change in gloss. The comparison was limited to the samples cleaned with the dry cloth, regardless of the type of graffiti removed (SP and XM).

**Figure 9:**
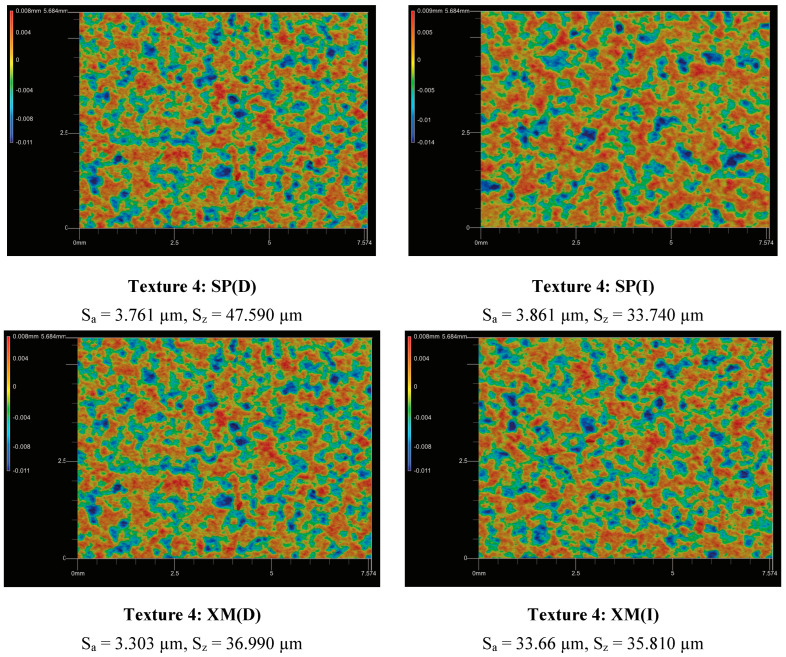
Topographic evaluation of surface (Texture 4) after graffiti removal, where (D)—dry cotton cloth and (I)—cotton cloth soaked in isopropyl alcohol (IPA).

Based on the measurement results, trend lines were determined for the relationship between the gloss changes and roughness (Ra, Rz, Rt and Rq). The trend line indicates the existence of a strong positive correlation of the relation G/G_I_ = f(R). As a result of the conducted research, a recommendation can be made that in order to assess the effectiveness of cleaning graffiti from painted surfaces, it is beneficial to prepare samples that are characterised by an appropriate roughness before painting. Based on the research results presented in this article, the texture should be characterised by a roughness described by the following roughness parameters: Ra < 2.15 µm, Rz < 10.32 µm, Rt < 13.45 µm and Rq < 2.58 µm. Such texture characteristics should guarantee that as a result of graffiti removal, any peeling of the paint surface (G/G_I_) will not exceed 1.10. Figure 9 presents the results of the topographic assessment of the surface (Texture 4) after cleaning.

The obtained results indicate that despite having anti-graffiti paint, which, due to its specification, can effectively facilitate the removal of graffiti stains, in the case of application on a surface with a developed texture, a positive result of removal attempts may not be achieved. This is important for products the surfaces of which cannot be highly smooth. For products made of plastics and polymer-based composites, especially products with a large surface, the lack of surface development is associated with a low-quality product. This applies, for example, to the interior furnishing of public transport vehicles, including bodies or seat casings. Based on the obtained research results, it was demonstrated that in the case of surfaces manufactured by injection using PA6 GF30, it is beneficial to carry out the painting process on surfaces the roughness of which after the injection process does not exceed R_a_ = 3.5 µm.

**Figure 10:**
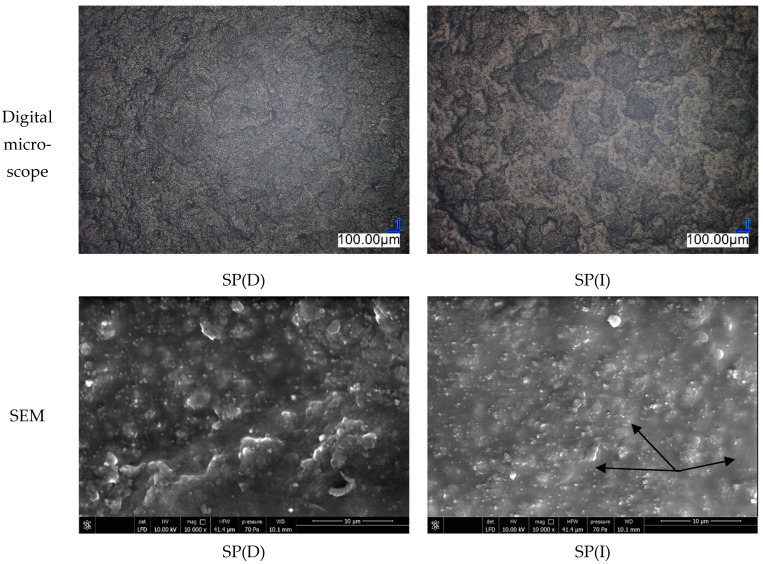
Surface morphology (Texture 4) after cleaning visualised with digital microscope (mag. 120×) and SEM (mag. 10,000×), where (D)—dry cotton cloth and (I)—cotton cloth soaked in isopropyl alcohol (IPA).

Observations of the surface morphology atter cleaning were carried out. The SEM observation results revealed evidence of the effect of plasticising agent and the deformation of the coat after the cotton cloth soaked in isopropyl alcohol (IPA) was applied (Figure 10).

**Figure 11:**
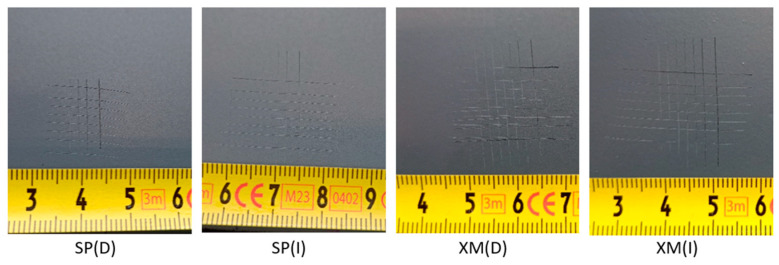
General view of cross-cuts in graffiti removal areas for Texture 4, where (D)—dry cotton cloth and (I)—cotton cloth soaked in isopropyl alcohol (IPA) (observation conditions: D65 light).

Adhesion of the coating to the substrate using the cross-hatch method was verified again in the graffiti removal areas (Figure 11). Positive results were confirmed.

**Figure 12:**
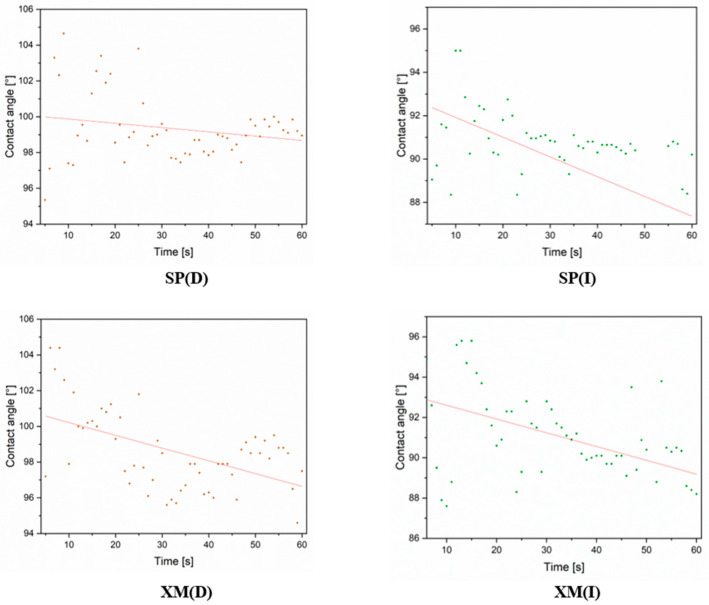
Dynamic water contact angle characterisation of cleaned surfaces, where (D)—dry cotton cloth and (I)—cotton cloth soaked in isopropyl alcohol (IPA).

The hydrophobic properties were also verified. The results of the dynamic characterisation of the water contact angle are presented in Figure 12.

In the case of the surfaces cleaned with dry cloth (SP(D) and XM(D)), a higher initial value was observed compared to those cleaned with cloth soaked in isopropanol (SP(I) and XM(I)). After 60 s of observation, the contact angle for the samples cleaned with dry cloth remained above 95°, and for the samples cleaned with isopropyl alcohol, the contact angle values dropped to below 90°. It is important to note that the hydrophobicity effect was retained.

## 4. Discussion

The use of wet painting technology for plastics enables a number of benefits to be achieved. Firstly, it offers an opportunity to reduce the risk of colour differences due to errors in the dye doping process (if the material is not mass-dyed by the supplier). Secondly, it is possible to use a larger amount of recyclate because there is no need to pay so much attention to possible deviations in the colour of the moulded part—an aspect of a circular economy [21,22,23], of course, assuming that all safety standards are met (including the automotive industry) [7,23,24,25]. Thirdly, paint coatings provide an opportunity to eliminate some surface defects, e.g., discolouration. Unfortunately, wet painting technology, especially when using higher gloss coatings, may highlight mould shape errors (e.g., technological pulls) or defects in the injection mould shaping elements. An important advantage is the ability to produce painted elements manufactured using injection technology special functional features (e.g., antibacterial properties, anti-graffiti) [26,27] without a significant risk of structural changes in the material that may have an adverse effect on the production technology and/or functional properties (e.g., a reduction in impact strength, deterioration of flammability properties). Coatings can also provide a protective barrier in the context of environmental conditions (humidity, UV radiation) [27,28].

The research presented in this article demonstrated that the use of the methodology for assessing the level of resistance to graffiti, taken from the ASTM D6578-13 standard, cannot necessarily be fully implemented in the automotive industry. According to the assessment methodology described in this article, the highest level of cleaning can be achieved when graffiti is completely removed using a dry cloth (Level 10). It is lower when using chemicals from mild detergent through alcohol (IPA), mineral spirits (e.g., kerosene) and then xylene and methyl ethyl ketone (MEK), with cleaning level orders 9, 8, 7, 6 and 5, respectively, provided that two conditions are met: (1) ΔE < 2.00 and (2) (G/G_I_) ∈ <0.90–1.10>. In the case of wet paints, especially those containing acrylic resins, the use of isopropanol turns out to be a bad solution. Cleaning acrylic in this manner leads to microfractures and cloudiness, compromising surface integrity. The influence of alcohol solvents on the properties of protective coatings containing acrylic resins has been the subject of many studies [18].

This article may be one of the first steps to formulate a standard for assessing anti-graffiti effects on polymer substrates in the automotive industry. The cleaning procedure should strive to standardise the assessment method in terms of not only the measurement methods and cleaning agents used, but also in terms of mechanical impact. When cleaning the samples with cotton cloth (with and without isopropanol), a standardised procedure should be applied, which relies on a defined pressure for the cotton cloth on the surface, and also a defined number of cycles (or movements) for rubbing away the graffiti. However, in order to remain in full compliance with the ASTM D6578-13 standard, it was ultimately decided to conduct the tests according to the requirements specified there: “Any trace of the contaminant should be rubbed vigorously until the stain is completely removed or until it can be visually determined that the traces can no longer be removed to a greater extent.” It should be noted that in this study, the direct pressure force and the number of cycles of hand movements during cleaning were not controlled, but the graffiti removal process was undertaken manually by the same operator in all the specimens. During the process, the stress was only controlled indirectly using analytical balance equipment to ensure the most repeatable conditions possible. It should be emphasised that by employing a manual surface cleaning method, full compliance with the ASTM D6578-13 standard was achieved. The use of a manual cleaning method also gives control over the generation of particles from the coating due to friction which are then be rubbed into the cleaned surface. The usage of a mechanical device (e.g., a tribological tester) working continuously in reciprocating or oscillating motion should be taken into account, and a methodology has to be proposed to avoid negative phenomena related to so-called third body abrasive wear of the surface [29].

It should also be noted that according to the marking scale specified in the standard, if the stain is completely removed using a dry cloth or a cloth soaked in isopropyl alcohol, the cleanability is set at cleaning level 10 or 8, respectively. An intermediate value is obtained for stains removed using a detergent. The publication did not present the results of re-cleaning using a detergent because the effects were poor. The lack of effective removal of the stains meant that taking measurements to characterise the surface was considered unjustified. The poor effectiveness of graffiti removal employing detergent can be explained by a significant reduction in the coefficient of friction. However, this conclusion indicates that the mechanical factor—friction—has a significant impact on the removal of the coating. This, in turn, leads to the conclusion that technological solutions in the form of anti-graffiti paints should be expected to have appropriate chemical (chemical stability), physical (hydrophobicity, hardness) and tribological (resistance to wear due to friction) properties.

The result of assessing the properties of the coatings and the cleaning effect may significantly depend on the above-mentioned factors. The results may also be different in the case of subsequent cycles of applying stains and removing them from the same areas of the test surfaces. The cleaning effect may also be influenced by the exposure of the coating material to environmental factors (e.g., UV-A). These issues will be the subject of further research. The cohesion of the surface needs to be verified as well.

## 5. Conclusions

Based on the performed investigations, the following conclusions are formulated:

1. The development of the texture of the painted surfaces has a negative impact on the process of the effective removal of graffiti (both caused by paint and marker). An initial high level of roughness may manifest itself in the lack of adequate effectiveness in removing graffiti in the roughness valleys and/or shearing or plastic deformation of the roughness peaks.
2. For the developed textures, the graffiti stains remain in the roughness valleys despite attempts to remove them. Increasing the difficulty of removing stains results in an intuitive need to increase the work necessary to remove the stains (greater number of cycles). This might increase the polishing effect and surface gloss.
3. Increasing the amount of work (greater pressure and/or more cycles) results in a polished surface. In the case of a relative change in gloss by more than 10%, the change in this parameter is noticeable to the naked eye—this phenomenon is obvious, but may be intensified if the wrong cleaning agent is applied (in our case, isopropyl alcohol acts as a plasticising agent for our paint coating).
4. The greater the roughness of the surface to be painted before and after painting, the greater the glossing (and smoothing) effect—this shows that the composition of the coating needs to be taken into account before initiating the cleaning procedure described in ASTM D6578-13. It is necessary to introduce a substitute with a potency similar to that of isopropyl alcohol (an agent classified somewhere between a mild detergent and mineral spirits).
5. Based on the obtained results, it is concluded that in order to ensure the proper anti-graffiti effect (in the sense of cleaning effectiveness and maintaining the colour and gloss parameters after cleaning), the texture should be characterised by the following roughness parameters: Ra < 2.15 µm, Rz < 10.32 µm, Rt < 13.45 µm and Rq < 2.58 µm.
6. Very good cohesion and high hydrophobic properties of the coating after cleaning are confirmed—negative effects of the cleaning agent are not found. The hydrophobic effect is maintained despite cleaning.

## Figures and Tables

**Table 1:** Average wettability for painted textures.

Texture	Wetting Angle [deg.]	S_a_ [µm]	S_z_ [µm]
0 (ref.)	87.67 (±5.16)	3.238	38.10
1	109.45 (±0.38)	24.397	135.90
2	105.83 (±0.30)	12.608	107.30
3	108.38 (±1.77)	79.708	391.70
4	108.81 (±0.97)	4.303	41.30

**Table 2:** Colour deviation and gloss measurement results after graffiti removal (G—gloss, G_I_—initial gloss, SP—spray paint, XM—xylene marker, D—dry, I—isopropanol, n/d—no data (ineffective cleaning)).

Texture	Colour and Gloss Comparison	After Painting/ before Cleaning	After Cleaning			
			SP (D)	SP (I)	XM (D)	XM (I)
0 (ref.)	ΔE	-	0.06	0.06	0.47	0.61
	G [%]	15.72	16.35	16.58	15.07	15.92
	G/G_I_	-	1.04	1.01	0.96	1.01
1	ΔE	-	0.89	0.63	n/d	1.24
	G [%]	2.77	3.41	3.59	n/d	4.65
	G/G_I_	-	1.23	1.30	n/d	1.68
2	ΔE	-	n/d	0.57	n/d	n/d
	G [%]	2.23	n/d	5.39	n/d	n/d
	G/G_I_	-	n/d	2.42	n/d	n/d
3	ΔE	-	0.35	0.39	n/d	0.77
	G [%]	2.42	2.71	5.51	n/d	4.06
	G/G_I_	-	1.12	2.28	n/d	1.68
4	ΔE	-	0.28	0.63	0.26	0.21
	G [%]	3.22	3.47	5.94	3.51	5.69
	G/G_I_	-	1.08	1.84	1.09	1.77

## Data Availability

Data are contained within the article.
